# Interrelationship of alcohol misuse, HIV sexual risk and HIV screening uptake among emergency department patients

**DOI:** 10.1186/1471-227X-13-9

**Published:** 2013-05-30

**Authors:** Alexis D Trillo, Roland C Merchant, Janette R Baird, George T Ladd, Tao Liu, Ted D Nirenberg

**Affiliations:** 1 Counseling, Education Leadership and School Psychology Department, Rhode Island College, Providence, RI, USA; 2 Department of Emergency Medicine, Alpert Medical School, Brown University, Providence, RI, USA; 3 Department of Epidemiology, Alpert Medical School, Brown University, Providence, RI, USA; 4 Department of Psychology, Chemical Dependency and Addiction Studies, Rhode Island College, Providence, RI, USA; 5 Department of Biostatistics, Alpert Medical School, Brown University, Providence, RI, USA; 6 Center for Statistical Sciences, Brown University, Providence, RI, USA; 7 Department of Psychiatry and Human Behavior, Alpert Medical School, Brown University, Providence, RI, USA; 8 Center for Alcohol and Addiction Studies, Brown University, Providence, RI, USA

**Keywords:** Emergency services, Hospital, Ethanol/blood, Questionnaires, Sexual behavior risk, HIV, Intervention

## Abstract

**Background:**

Emergency department (ED) patients comprise a high-risk population for alcohol misuse and sexual risk for HIV. In order to design future interventions to increase HIV screening uptake, we examined the interrelationship among alcohol misuse, sexual risk for HIV and HIV screening uptake among these patients.

**Methods:**

A random sample of 18-64-year-old English- or Spanish-speaking patients at two EDs during July-August 2009 completed a self-administered questionnaire about their alcohol use using the Alcohol Use Questionnaire, the Alcohol Use Disorders Identification Test (AUDIT), and the HIV Sexual Risk Questionnaire. Study participants were offered a rapid HIV test after completing the questionnaires. Binging (≥ five drinks/occasion for men, ≥ four drinks for women) was assessed and sex-specific alcohol misuse severity levels (low-risk, harmful, hazardous, dependence) were calculated using AUDIT scores. Analyses were limited to participants who had sexual intercourse in the past 12 months. Multivariable logistic regression was used to assess the associations between HIV screening uptake and (1) alcohol misuse, (2) sexual risk for HIV, and (3) the intersection of HIV sexual risk and alcohol misuse. Adjusted odds ratios (AORs) with 95% confidence intervals (CIs) were estimated. All models were adjusted for patient demographic characteristics and separate models for men and women were constructed.

**Results:**

Of 524 participants (55.0% female), 58.4% identified as white, non-Hispanic, and 72% reported previous HIV testing. Approximately 75% of participants reported drinking alcohol within the past 30 days and 74.5% of men and 59.6% of women reported binge drinking. A relationship was found between reported sexual risk for HIV and alcohol use among men (AOR 3.31 [CI 1.51-7.24]) and women (AOR 2.78 [CI 1.48-5.23]). Women who reported binge drinking were more likely to have higher reported sexual risk for HIV (AOR 2.55 [CI 1.40-4.64]) compared to women who do not report binge drinking. HIV screening uptake was not higher among those with greater alcohol misuse and sexual risk among men or women.

**Conclusions:**

The apparent disconnection between HIV screening uptake and alcohol misuse and sexual risk for HIV among ED patients in this study is concerning. Brief interventions emphasizing these associations should be evaluated to reduce alcohol misuse and sexual risk and increase the uptake of ED HIV screening.

## Background

Alcohol use has been linked to sexual risk [1-7] and unsafe sexual practices, including inconsistent condom use [8-14] and multiple sexual partners [9,14,15] among high-risk groups such as college students, commercial sex workers, and injection-drug users. Binge drinking, defined by the National Institute on Alcohol Abuse and Alcoholism (NIAAA) as ≥ four drinks for women and ≥ five drinks for men on one occasion [16], has been associated with having either syphilis, gonorrhea or trichomoniasis (AOR 1.56 [CI 1.00-2.41]) [13]. In a large-scale, cross-sectional study of 41,073 participants across the US, bingers were 1.77 times more likely to engage in human immunodeficiency virus (HIV) risk behaviors (including injection-drug use, exchange of sex for money/drugs, and anal sex without condoms) than non-bingers [17]. In a review of research conducted in eight countries, alcohol use was considered a facilitator of sexual risk behaviors, such as inconsistent condom use and multiple sexual partners [18]. Furthermore, in a cross-sectional study of 1,268 men and women in Botswana, there was a three-fold increase chance of having unprotected sex and multiple sex partners in the past month among women and men with heavy alcohol consumption (>14 drinks/week for women and >21 drinks/week for men), compared to moderate alcohol consumers [14].

Not only is alcohol misuse associated with sexual risk, it also has been shown to be related to HIV acquisition. In a cross-sectional study of 2,374 sexually active adults in rural Uganda, Mbulaiteye et al. reported a significant association between alcohol consumption and HIV seropositivity in that individuals with a history of any alcohol use had twice the prevalence of HIV infection when compared to individuals without a history of alcohol use (10% vs. 5%; p < 0.001) [19]. In a meta-analysis of 20 African studies assessing the relationship between alcohol use (defined as daily consumption of greater than three drinks per occasion) and HIV infection, Fisher et al. observed that drinkers are at over 70% higher risk for acquiring or having HIV than non-drinkers [20]. Zablotska et al. conducted a longitudinal study among 14,875 individuals in Uganda and found that the incidence of HIV when one partner consumed alcohol before sex was aIRR 1.67 (CI 1.17–2.40) among men, and aIRR 1.40 (CI 1.02–1.92) among women, and when both partners consumed alcohol the incidence was aIRR 1.58 (CI 1.13–2.21) among men, and aIRR 1.81 (CI 1.34–2.45) among women [21].

The intersection of alcohol misuse and HIV is an important topic in the emergency department (ED) setting. At least 26% of ED patients meet NIAAA criteria for “at-risk” drinking [22], defined as heavy or problematic alcohol use that may lead to an array of negative consequences, including social, physical, psychological, legal and financial problems [23]. Selected US ED patient populations also have been shown to have a relatively high prevalence of undiagnosed HIV infection [24-30]. A few studies have found high proportions of ED patients who engage in HIV risk behaviors. In a randomized, controlled trial conducted at a Boston ED, Bernstein et al. observed that among a high-risk patient population of substance users, 70% of patients reported engaging in sex without a condom in the past 30 days, and 36% reported having sex without a condom with casual or transactional sex partners [31]. In a large-scale study involving 29 EDs across France, 40.2% of 11,356 patients reported multiple sexual partners within the past 12 months [32]. Alpert et al., conducted a cross-sectional study at a New York ED with 1,744 participants, of which 37.6% reported engaging in one or more HIV risk behaviors, such as injection-drug use, male-to-male sex, sex with partners who have a history of drug use and a sexually transmitted infection (STI) or HIV, transactional sex, and a history of ten or more sex partners in the past year [24]. Furthermore, among participants who reported only one sexual partner, a seemingly “low risk” population, 15.0% of women and 4.6% of men reported that their usual sexual partner had other concurrent partners in the past year [33]. Of 557 randomly selected participants at an urban northeastern US ED, 12.8% of men and 5.8% of women reported injection-drug use, 43.6% of men and 50.2% of women reported having unprotected vaginal/anal sex with multiple sexual partners, 4.7% of men reported having had unprotected anal sex with men and 4.3% of women reported having unprotected sex with men who had sex with another man in the past ten years [34].

Studies have demonstrated a growing interest in conducting HIV screening in EDs, but uptake of HIV screening has varied across US EDs (13.0% to 99.8%) due to differences in populations studied, methods employed, and interventions or incentives offered [25-30,35-61]. HIV screening uptake in EDs has been associated with the perception of personal risk for acquiring HIV, as well as varying by patient demographic characteristics [43,62]. One of the most common reasons for declining HIV screening is lack of perception of risk for HIV infection [25,28,30,32,35,39-41,50,51],[53,58,61-64]. Due to the high prevalence of reported sexual risk and alcohol misuse by ED patients, many techniques have been utilized, with mixed results, to increase uptake of HIV screening, including opt-out HIV screening [36,45,54-57,60,61,65], financial incentives [66], ED staff or clinician-initiated testing [51,54,67], oral fluid sampling for testing [53], prevention counseling [64], and video or computer-based interventions [43,68,69]. Although a number of studies have examined alcohol misuse, HIV risk, and HIV screening, there is a paucity of research on the intersection of these issues. One approach to improve HIV screening uptake may be to combine alcohol-related and HIV risk interventions in order to increase self-perceived risk and potentially increase acceptance of screening among ED patients.

Before creating such interventions to improve HIV screening uptake and reduce HIV risk and alcohol misuse, the interrelationships among alcohol misuse, HIV risk and uptake of HIV screening in the absence of interventions among ED patients need to be established. Our interests in this study were to examine the intersection of alcohol misuse and sexual risk for HIV in its relationship to HIV screening uptake among ED patients. In particular, our objectives were to determine the association between: (1) reported alcohol misuse and HIV sexual risk behaviors; (2) reported alcohol misuse and HIV screening uptake; and (3) reported sexual risk and HIV screening uptake in the absence of any interventions. We hypothesized that those who reported greater alcohol use and sexual risk for HIV would be more inclined to accept HIV screening.

## Methods

### Study design and setting

From July 2009 to August 2009, 18- to 64-year-old ED patients were randomly selected for inclusion in this study. This investigation had two components: (1) a cross-sectional study examining the prevalence of alcohol misuse and HIV sexual risk among ED patients, (2) and an examination of opt-in HIV screening in this population. The study was conducted at two academic EDs (Rhode Island Hospital and The Miriam Hospital) located in Providence, Rhode Island, that are affiliated with the Alpert Medical School of Brown University. Rhode Island Hospital is a level 1 trauma center, receiving over 100,000 annual adult patient visits, and the Miriam Hospital is a community hospital, receiving over 55,000 annual adult patient visits. The Rhode Island Hospital Institutional Review Board approved this study. Verbal consent was obtained for the cross-sectional component of the study, and written consent was obtained for the HIV testing component.

### Selection of participants

Similar to previous studies conducted in these EDs [44,70], this study obtained a representative sample of participants by approaching randomly selected ED patients for study inclusion on randomly selected dates and shifts. Fifty-seven dates during an eight-week period (July 2009-August 2009) were randomly selected; all days of the week had an equal chance of selection. For these 57 dates, 72 shifts were randomly selected using a weighting scheme corresponding to patient ED volume during a typical 24-hour period (40% of shifts were from 8:00 am-4:00 pm, 50% from 4:00 pm-midnight and 10% from midnight-8:00 am). On those shifts, 80% of patients in the ED were randomly selected for possible inclusion in the study. This random selection of patients was based upon their ED medical record number, the last two digits of which were matched to numbers randomly selected by a computer program (http://www.random.org). Patient eligibility for the study was assessed for these randomly selected patients by a research assistant (RA) through a review of their ED medical record and confirmation of their eligibility through an in-person interview. Patients were study eligible if they were: age 18-64-years; English- or Spanish-speaking; not critically ill or injured; not prison inmates, not under arrest, or undergoing home confinement; not presenting for an acute psychiatric illness; not intoxicated; not HIV infected; not participating in an HIV vaccine trial, and did not have a physical disability or mental impairment that prevented them from providing consent for participating in the study. No incentives were offered to participants. ED staff members were not permitted to encourage or refer patients to be in the study.

### Study questionnaire content and administration

Participants were interviewed by the RA about their demographic characteristics (age; race/ethnicity; partner status; insurance status; and education level) and history of ever being tested for HIV through blood donation, screening, or diagnostic testing; and time elapsed since blood donation or HIV testing. These demographic and HIV testing history questions were developed for and used in previous studies [34,62,70]. Participants completed self-administered confidential questionnaires regarding the quantity and frequency of their alcohol use, severity of their alcohol use, and sexual risk for HIV on tablet computers using the Questionnaire Development System (QDS) (NOVA Research Company, Bethesda, MD). The survey questionnaires were finalized in English, translated into Spanish then back translated into English to ensure translation accuracy using accepted techniques [71-74]. An English-language copy of the questionnaires is provided in the supplementary material (see Additional file 1). The questionnaires were available in English or Spanish and were completed by participants while they awaited medical care.

Alcohol misuse and disorder severity during the past 12 months was measured through the ten-item Alcohol Use Disorders Identification Test (AUDIT), developed by the World Health Organization [23]. The AUDIT is a well-established alcohol misuse screening and severity instrument for the ED and other settings with excellent reliability and validity [23,75-77]. Quantity and frequency of alcohol use during a typical month in the past 12 months was assessed by a six-question survey (The Alcohol Use Questionnaire) developed by the study authors for the purpose of this study and based on research questions used in previous studies [23,78,79]. Through this questionnaire, participants were queried about the number of days they spent drinking in a typical month, the number of drinks consumed on a typical day, their alcohol beverage choice, the most number of drinks consumed on one occasion, and the number of days spent engaging in binge drinking in a typical month. Binge drinking was assessed using NIAAA recommended definitions [16]. Per these recommendations, male participants were asked on how many days they consumed five or more drinks and female participants were asked on how many days they consumed four or more drinks on one occasion in a typical month during the past 12 months. The Alcohol Use Questionnaire complemented the AUDIT in that participants were asked for specifics regarding the number of days they drank alcohol and the amounts used; whereas, the AUDIT employs categorical designations as the responses for these questions. Further, the Alcohol Use Questionnaire permitted sex-specific responses for binge drinking. A Chronbach’s Alpha analysis showed an acceptable level of internal consistency (α = 0.80), and a strong correlation between relevant questions from the Alcohol Use Questionnaire and total AUDIT scores (ρ = 0.66-0.73) in the population included in this study.

Participants also completed the HIV Sexual Risk Questionnaire, consisting of multiple-choice, closed-response questions about their reported HIV sexual risk behaviors. The questions were derived from the CDC National HIV Behavioral Surveillance (NHBS) System survey and adapted through cognitive testing for this study and previous studies [34,43,70,80]. This questionnaire consisted of primary questions with associated sequences of follow-up questions, which would only appear if the participant answered affirmatively to the primary questions. The number of questions answered by each participant was dependent upon their reported HIV sexual risk behaviors in the past 12 months. Sexual risk for HIV was assessed separately for males and females due to the different types of sexual risks they engage in; therefore, the questions were sex-specific. Accordingly, females were asked questions regarding anal and/or vaginal sex with males and males were asked about anal and/or vaginal sex with females, and anal sex with males. A Chronbach’s Alpha analysis confirmed a strong level of internal consistency for these questions among females (α = 0.90) and among males (α = 0.84).

In the HIV Sexual Risk Questionnaire, participants were queried about HIV sexual risk behaviors in the past 12 months by sexual partner type as by CDC-recommended definitions [80]. Sexual partner types were: (1) main partner(s), defined for participants as “men or women you felt committed to, such as boyfriends or girlfriends, husbands or wives, significant others or life partners”; (2) casual partner(s), defined as “men or women you had sex with but did not feel committed to”; and (3) exchange partner(s), defined as “men or women you gave money, drugs, or other things to pay for sex, or men or women you had sex with so they would give you money, drugs or other things.” Participants were asked to report the type(s) of sexual partners (main, casual and exchange partner), unprotected sex (anal and/or vaginal sexual intercourse) with these partners and the number of sexual partners by partner type according to their partner’s history of injection drug use, sexually transmitted diseases (STDs), and HIV status. According to each partner type, participants were asked how many of their sexual partners they (1) knew or (2) how many they were unsure if they had HIV, (3) were injection-drug users, or (4) had an STD. In addition, females also were asked the number of male sexual partners they knew and also the number of male sexual partners they were unsure about had had sex with other males.

The study authors developed three additional questions regarding the intersection of alcohol misuse and HIV sexual risk behaviors. The questions asked participants if they had ever had sex while intoxicated, regretted ever having had sex while intoxicated, and if they were ever unsure if they had sex while intoxicated in the past 12 months. A Chronbach’s Alpha analysis confirmed an acceptable level of internal consistency for these questions among female drinkers (α = 0.73) and among male drinkers (α = 0.67).

### HIV screening

At the conclusion of the study, participants were asked by the RA if they would like to be tested for HIV using a free rapid HIV test (opt-in HIV screening). Participants were informed that HIV screening was voluntary, involved a rapid HIV test using a finger stick of blood, and that results would be provided to them within 20–30 minutes. The OraQuick *ADVANCE*® Rapid HIV-1/2 Antibody Test was performed (http://www.orasure.com). No incentives for HIV screening were offered and participants were not provided with an intervention or encouragement to be tested. RAs were blinded to participant’s alcohol use or misuse and HIV sexual risk history. Participants were not informed at the start of the study that they would be offered an HIV test. Uptake of HIV screening was an outcome measure for the study. As such, the relationship of participant reported alcohol misuse and sexual risk for HIV to uptake of HIV screening was assessed. A follow-up question asked participants about the main reasons why they accepted or declined screening. All participants who agreed to be tested were offered HIV risk-reduction counseling in English or Spanish. All RAs were bilingual in English and Spanish. The RAs also completed a state-sponsored training program on HIV counseling and screening as well as a training program on rapid HIV screening. No patients tested positive for HIV in this study.

### Data analysis

All statistical analyses were conducted using STATA 11 (Stata Corp., College Station, TX). Participant screening and enrollment were summarized and diagramed per the Strengthening the Reporting of Observational Studies in Epidemiology (STROBE) recommendations [81]. Demographic characteristics, HIV screening history, alcohol misuse, sexual risk for HIV and reasons why participants accepted or declined HIV testing were also summarized by sex. Data are reported using mean, median, standard deviation (SD), and interquartile range (IQR) where appropriate.

The percentage of days spent drinking in one month was calculated by dividing the number of days spent drinking in one month by 31 days. Presence of binge drinking was determined by the aforementioned cutoffs of ≥ five drinks for men and ≥ four drinks for women [16]. The percentage of days spent binging was calculated by dividing the number of days spent binging by the number of total days spent drinking in one month. Percentage of days spent drinking in one month and percentage of days spent binging were converted into four levels (0-24%, 25-49%, 50-74%, 75-100%) because values were not normally distributed and to aid in ease of interpretation. For the AUDIT, participants were classified into at-risk drinking levels as recommended by Babor, Biddle, Saunders and Monteiro [23]. For men, a score of < eight for men and < six for women indicated a low-risk drinking level [82]. A score ≥ eight and ≤ 15 for men and ≥ six and ≤ 13 for women indicated a hazardous drinking level [83]. A score ≥ 16 and ≤ 19 for men and ≥ 14 and ≤ 17 for women indicated a harmful drinking level. A score of ≥ 20 for men and ≥ 18 for women determined a dependent drinking level.

Based upon their HIV Sexual Risk Questionnaire responses, all participants who reported no sexual intercourse within the past 12 months were eliminated from the study analysis. HIV sexual risk scores were calculated for those who reported having sexual intercourse within the past 12 months. Points for HIV sexual risk scores were assigned based on the reported type of sexual partner. We assigned one point for participants who reported having unprotected sex with their main partner, and two points each for having unprotected sex with a casual partner and/or with an exchange partner. Additional points were assigned based upon the number of unprotected sexual partners and upon characteristics of the participant’s sexual partners (e.g. HIV status, injection- drug use and history of STD infection). The highest possible score was 209 for females and 514 for males. HIV sexual risk scores were transformed into a logarithmic scale because the scores were not normally distributed. The log of HIV sexual risk scores were divided into tertiles for ease of interpretation. Tertiles were based upon the distribution of the log of the HIV sexual risk scores, and not an even distribution of the participants. As such, they were grouped according to three levels of reported HIV risk, and hence the sizes of the groups were not equal.

Multivariable and univariable regression analyses were used to assess for relationships between (1) log of HIV sexual risk scores in tertiles and alcohol misuse, (2) HIV screening uptake and alcohol misuse, (3) HIV screening uptake and sexual risk for HIV, and (4) HIV screening uptake and the intersection of HIV sexual risk and alcohol misuse (sex while intoxicated, regret ever having had sex while intoxicated, and unsure if ever had sex while intoxicated). Ordinal logistic regression modeling was performed for analyzing associations between the log of HIV sexual risk levels in tertiles and whether participants drink or not; percentage of days spent drinking and binging in one month; AUDIT at-risk drinking levels; and whether participants binge or not. Logistic regression modeling was used to assess the outcome of HIV screening uptake as related to (1) alcohol misuse, (2) the log of HIV sexual risk levels in tertiles; and (3) the intersection of alcohol misuse and sexual risk for HIV. Based upon responses for declining HIV screening, logistic regression modeling assessed the outcome of participant’s perception of not being at risk for an HIV infection and drinking and binging status among all participants and drinkers. Goodness-of-fit of the logistic regression models was confirmed by Hosmer-Lemeshow analyses. Adjusted odds ratios (AORs) with corresponding 95% confidence intervals (CIs) were estimated. Multivariable regression models were adjusted for participant demographic characteristics (age, race/ethnicity, partner status, insurance status and education level). Our previous research indicated that demographic characteristics are important correlates for uptake of HIV screening, hence we adjusted for our main effects for these confounding variables [43,62]. All analyses were considered significant at an α level of 0.05, with no adjustments for multiple comparisons.

## Results

### Participant enrolment and demographic characteristics

**Figure 1:**
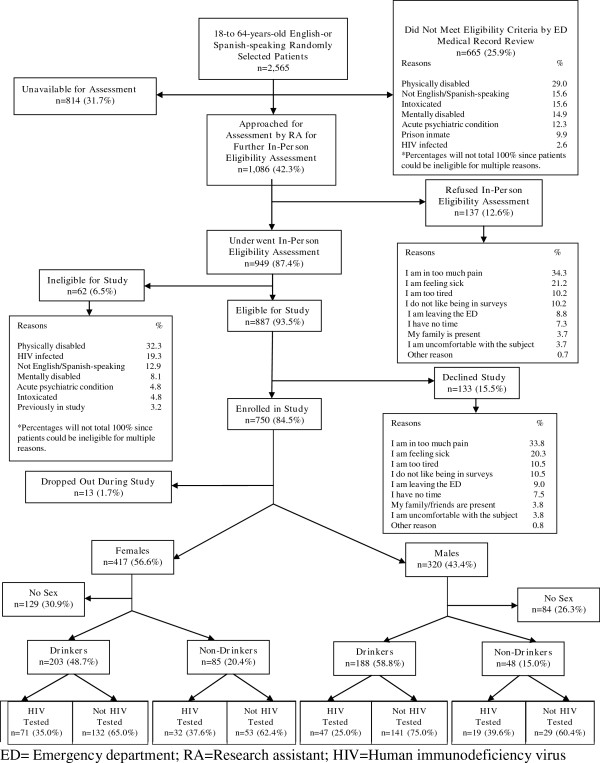
Eligibility and enrolment flow diagram.

During the two-month study period, 2,565 randomly selected 18-64-year-old English or Spanish-speaking ED patients were assessed for study eligibility. Of the 887 study eligible ED patients, 750 enrolled in the study. Figure 1 depicts the results of eligibility assessments, the major reasons for study ineligibility and for accepting and declining study participation. As shown, 28.9% of participants reported not having sexual intercourse in the past 12 months, which left 524 participants who reported some sexual risk for HIV, and who constituted the final study sample used for these analyses.

Table 1 shows the participant demographic characteristics and HIV screening history by sex. Of all 524 participants, 55.0% were female. The median age was 39 (IQR, 27–50 years) for males and 34 (IQR, 26.5-45.5) for females. For both males and females, most participants were white, non-Hispanic, had private health insurance, had never been married, and had 12 years or more of formal education. Seventy-two percent of participants reported having been tested for HIV (76.4% of women, 66.5% of men). The majority of participants in this study had been tested for HIV more than five years ago, had never donated blood, and had been tested for HIV but not as part of a blood donation.

**Table 1:** Participant demographic characteristics and HIV screening history

	**Females**	**Males**
	***n = 288***	***n = 236***
***Demographic characteristics***		
**Median age, years (Interquartile Range)**	34 (26.5-45.5)	39 (27–50)
	***%***	***%***
**Ethnicity/Race**		
White, non-Hispanic	53.1	64.8
White, Hispanic	22.9	10.2
Black, non-Hispanic	10.4	14.0
Black, Hispanic	8.4	8.0
Other	5.2	3.0
**Health insurance status**		
Private	48.3	48.7
Governmental	35.4	25.9
None	16.3	25.4
**Partner status**		
Never married	32.6	36.0
Divorced/Widowed/Separated	13.6	12.3
Married	35.4	42.0
Unmarried couple	18.4	9.7
**Years of formal education**		
Grades 1-11	16.3	18.6
Grade 12/GED	32.0	38.2
College/Graduate studies	51.7	43.2
***HIV testing history***		
**Ever tested for HIV (Not part of a blood donation)**		
Previously	76.4	66.5
Never	22.9	33.1
Don’t know	0.7	0.4
**Time elapsed since last HIV test**	***n = 220***	***n = 157***
>5 years	26.4	27.4
>2 years but ≤5 years	18.2	18.5
>1 year but ≤2 years	16.8	17.8
>6 months but ≤1 years	20.0	17.2
≤6 months	18.2	19.1
Don’t know	0.4	0.0
**Ever donated blood**	***n = 288***	***n = 236***
Yes	39.2	44.5
No	60.8	55.5
**Time elapsed since last blood donation**	***n = 113***	***n = 105***
>5 Years	37.2	54.3
>2 years but ≤5 years	14.2	19.0
>1 year but ≤2 years	15.9	8.6
>6 months but ≤1 years	16.8	7.6
≤6 months	15.0	10.5
Don’t know	0.9	0.0
**History of any HIV test**	***n = 288***	***n = 236***
Tested, but not part of a blood donation	45.8	35.6
Tested as part of a blood donation	8.7	13.6
Tested and donated blood	30.6	30.9
No known HIV test	14.6	19.9
Don’t know	0.3	0.0

GED = General equivalency diploma; HIV = Human immunodeficiency virus.

### Participant alcohol misuse, sexual risk for HIV

Table 2 depicts participant alcohol misuse and sexual risk for HIV by sex. Approximately 75% of participants reported drinking alcohol within the past 30 days.

**Table 2:** Participant alcohol misuse, HIV risk and the intersection of HIV risk and alcohol misuse

	**Females**	**Males**
	**%**	**%**
***Alcohol use/misuse***		
**Participants who drink alcohol**	***n = 288***	***n = 236***
No	29.5	20.3
Yes	70.5	79.7
**Percentage of days spent drinking alcohol in one month**		
0 - 24%	38.4	21.8
25 - 49%	25.1	14.9
50 - 74%	20.2	30.3
75 - 100%	16.3	33.0
**At-risk drinking levels based on AUDIT scores**	***n = 203***	***n = 188***
Low-risk	68.0	62.8
Hazardous	26.1	26.1
Harmful	2.5	3.7
Dependence	3.4	7.4
**Participants who binge**		
No	40.4	25.5
Yes	59.6	74.5
**Percentage of days spent binging in one month**	***n = 121***	***n = 140***
0 - 24%	8.3	7.1
25 - 49%	32.2	20.0
50 - 74%	33.1	39.3
75 - 100%	26.4	33.6
***HIV risk***	***n = 288***	***n = 236***
**Log of HIV risk score**		
Tertile 1	61.5	52.5
Tertile 2	17.0	20.4
Tertile 3	21.5	27.1
**Partner status**		
No partner	0.4	0.4
Main partner only	86.1	68.2
Casual partner +/− exchange partner or main partner	13.5	31.4
**Unprotected sex by partner type**	***n = 178***	***n = 150***
Main partner only	84.8	70.7
Casual partner +/− exchange partner or main partner	15.2	29.3
***HIV risk and alcohol misuse***	***n = 203***	***n = 188***
**Sexual intercourse while intoxicated**		
Never	74.9	63.8
Ever	25.1	36.2
**Regret ever having had sexual intercourse while intoxicated**		
Never	87.7	84.6
Ever	12.3	15.4
**Unsure if had sexual intercourse while intoxicated**		
Never	95.0	93.6
Ever	5.0	6.4

HIV = Human immunodeficiency virus; AUDIT = Alcohol Use Disorders Identification Test.

Thirty-three percent of males and 16.3% of females reported spending 75 – 100% days of the month drinking alcohol. The majority of participants fell within the low-risk drinking level based on AUDIT scores; however, 74.5% of males and 59.6% of females reported binging, and 26.1% of both females and males would be classified as drinking at hazardous levels. The majority of participant’s log HIV sexual risk scores fell within the first tertile level. The majority of participants, 86.1% of females and 68.2% of males, reported having only a main partner. Among drinkers, 88.6% of females and 79.8% of males reported having unprotected sex, and among non-drinkers, 80.0% of females and 80.9% males reported having unprotected sex in the past 12 months. Approximately 36.2% of males and 25.1% of females reported having sex while intoxicated, 15.4% of males and 12.3% of females regretted ever having had sex while intoxicated and 6.4% of males and 5.0% of females were unsure if they had sex while intoxicated in the past 12 months.

### Relationship of sexual risk for HIV to alcohol misuse

In Table 3, results of multivariable logistic regression analyses demonstrate, for both males and females, drinking status (whether participants drink or not), was strongly associated with an increase in sexual risk for HIV. For female drinkers, a greater percentage of days spent drinking and binging, reaching hazardous and harmful AUDIT levels and binging status were associated with increased sexual risk for HIV among females. For male drinkers, a higher percentage of days spent drinking and hazardous and dependent AUDIT levels were associated with increasing sexual risk for HIV.

**Table 3:** Multivariable logistic regression analyses comparing log of HIV sexual risk score and alcohol misuse, and HIV screening uptake and alcohol misuse

	***Females***		***Males***	
	**Log of HIV sexual**	**HIV testing uptake**	**Log of HIV sexual**	**HIV testing uptake**
	**Risk score**^**a**^		**Risk score**^**a**^	
Among all participants	n = 288		***n = 236***	
**Participants who drink alcohol**	AOR (95% CI)*	AOR (95% CI)*	AOR (95% CI)*	AOR (95% CI)*
No	**Reference**		**Reference**	
Yes	2.78 (1.48-5.23)	0.95 (0.52-1.72)	3.31 (1.51-7.24)	0.58 (0.26-1.29)
***Among alcohol drinkers***	***n = 203***		***n = 188***	
**Percentage of days spent drinking alcohol in one month**				
0 - 24%	**Reference**		**Reference**	
25 - 49%	1.92 (0.88-4.18)	1.09 (0.45-2.67)	0.57 (0.19-1.70)	2.17 (0.50-9.43)
50 - 74%	3.27 (1.47-7.28)	0.57 (0.22-1.43)	1.88 (0.82-4.32)	3.08 (0.95-10.01)
75 - 100%	4.84 (2.00-11.69)	1.47 (0.56-3.86)	2.51 (1.07-5.89)	1.87 (0.59-6.00)
**At-risk drinking levels based on AUDIT scores**				
Low-risk	**Reference**		**Reference**	
Hazardous	3.85 (1.89-7.85)	1.10 (0.50-2.41)	3.45 (1.72-6.92)	0.59 (0.23-1.53)
Harmful	10.64 (1.70-66.53)	8.81 (0.88-87.85)	0.60 (0.10-3.48)	2.70 (0.47-15.65)
Dependence	∞	6.52 (0.69-61.91)	9.61 (2.45-37.69)	3.16 (0.75-13.25)
**Participants who binge**^**b**^				
No	**Reference**		**Reference**	
Yes	2.55 (1.40-4.64)	0.99 (0.51-1.93)	1.84 (0.91-3.72)	1.61 (0.63-4.12)
***Among bingers***	***n = 121***		***n = 140***	
**Percentage of days spent binging out of days spent drinking alcohol**				
0 - 24%	**Reference**		**Reference**	
25 - 49%	2.62 (0.55-12.53)	0.94 (0.17-5.32)	2.00 (0.39-10.29)	2.03 (0.25-16.36)
50 - 74%	3.28 (0.68-15.88)	0.99 (0.18-5.65)	1.82 (0.39-8.43)	1.48 (0.20-10.89)
75 - 100%	9.25 (1.84-46.43)	0.66 (0.11-3.79)	2.24 (0.49-10.29)	1.76 (0.25-12.46)

^*^All multivariable models were adjusted for demographic characteristics.

^a^Ordinal logistic regression modeling was performed for outcome of tertile of HIV Sexual Risk Score.

^b^ ≥4 drinks for women and ≥5 drinks for men on one occasion as defined by NIAAA (NIAAA, 2004).

HIV = Human immunodeficiency virus; AOR = Adjusted odds ratio; CI = Confidence interval; AUDIT = Alcohol Use Disorders Identification Test. ∞ = not estimable.

### HIV screening uptake

As shown in Table 3, multivariable logistic regression was performed to assess the relationship between HIV screening uptake and alcohol misuse. There was no relationship found for both males and females. Table 4 illustrates the results of the multivariable logistic regression analyses examining the association between HIV testing uptake and log of HIV sexual risk, stratified by alcohol misuse. No relationships were identified among all participants, participants who drink alcohol and do not drink alcohol, for both males and females.

**Table 4:** Multivariable logistic regression analyses comparing HIV screening uptake and log of HIV sexual risk score, stratified by alcohol use

	**HIV testing uptake**	
	AOR (95% CI)*	
***Log of HIV sexual risk score***	***Females***	***Males***
**All participants**	***n = 288***	***n = 236***
Tertile 1	**Reference**	**Reference**
Tertile 2	0.84 (0.40-1.77)	0.79 (0.30-2.09)
Tertile 3	0.98 (0.51-1.89)	1.62 (0.74-3.53)
**Participants who drink alcohol**	***n = 203***	***n = 188***
Tertile 1	**Reference**	**Reference**
Tertile 2	1.00 (0.43-2.33)	1.18 (0.41-3.43)
Tertile 3	1.01 (0.46-2.21)	1.80 (0.72-4.47)
**Participants who do not drink alcohol**	***n = 85***	***n = 48***
Tertile 1	**Reference**	**Reference**
Tertile 2	0.42 (0.03-5.71)	∞
Tertile 3	0.26 (0.03-2.14)	11.74 (0.05-2994.57)

*All multivariable models were adjusted for demographic characteristics. HIV = Human immunodeficiency virus; AOR = Adjusted odds ratio; CI = Confidence interval. ∞ = not estimable.

Univariable logistic regression and multivariable logistic regression analyses were conducted to assess the associations between HIV screening uptake and the intersection of sexual risk for HIV and alcohol misuse, as shown in Table 5. Univariable regression analyses revealed a strong relationship between HIV screening uptake, regretting ever having had sex while intoxicated, and unsure if ever had sex while intoxicated among female drinkers. For male drinkers, a relationship was found between HIV screening uptake, sex while intoxicated and unsure if ever had sex while intoxicated. However, when adjusting for demographic characteristics in the multivariable logistic regression analyses, there were no relationships found for both males and females.

**Table 5:** Univariable and multivariable logistic regression analyses comparing HIV screening uptake and the intersection of HIV sexual risk and alcohol misuse

	**HIV testing uptake**			
	**Univariable analysis**		**Multivariable analysis**	
	**UOR (95% CI)**		AOR (95% CI)*	
	***Females***	***Males***	***Females***	***Males***
**Sexual intercoursewhile intoxicated**	***n = 203***	***n = 188***	***n = 203***	***n = 188***
Never	**Reference**		**Reference**	
Ever	0.81 (0.41-1.59)	2.59 (1.32-5.09)	0.45 (0.21-1.00)	2.21 (0.98-4.98)
**Regret ever having had sexual intercourse while intoxicated**				
Never	**Reference**		**Reference**	
Ever	2.70 (1.15-6.32)	1.43 (0.60-3.41)	1.81 (0.70-4.66)	1.12 (0.34-3.14)
**Unsure if had sexual intercourse while intoxicated**				
Never	**Reference**		**Reference**	
Ever	4.78 (1.20-19.10)	3.29 (1.01-10.76)	2.75 (0.62-12.29)	2.49 (0.64-9.73)

*All multivariable models were adjusted for demographic characteristics. HIV = Human immunodeficiency virus; UOR = Unadjusted odds ratio; AOR = Adjusted odds ratio; CI = Confidence interval.

### Reasons for accepting or declining HIV screening

We examined factors related to reasons why participants accepted or declined HIV screening in the ED. Of those who agreed to screening, among women, 33.7% of drinkers and 25.5% of non-drinkers cited convenience as the most common reason why they accepted screening. Among men, 26.9% of drinkers and 32.1% of non-drinkers who accepted screening cited “because you asked” as the most common reason. Of the participants who declined screening, among women 51.7% of drinkers and 60% of non-drinkers, and among men 46.9% of drinkers and 63% of non-drinkers cited they did not believe they were at risk as the most common reason for not being screened. In examining the relationship between alcohol misuse and acceptance of screening, alcohol drinkers were just as likely as non-drinkers to say that they were not at risk for HIV among males (AOR 2.33 [0.89-6.11]) and females (AOR 0.83 [0.35-1.94]). Bingers were just as likely as non-bingers to say that they were not at risk for HIV among males (AOR 1.50 [0.62-3.64]) and females (AOR 1.03 [0.41-2.63]).

## Discussion

Previous studies have noted a high prevalence of reported alcohol misuse, at-risk drinking and sexual risk for HIV among US ED patients [22,33,34,70,84,85]. Among participants in this study, too, there was a high prevalence of reported alcohol misuse and sexual risk for HIV. Our study results were consistent with our hypothesis that there was a relationship between reported alcohol misuse and reported sexual risk for HIV among participants who consume alcohol. However, we did not observe a relationship between reported alcohol misuse and HIV screening uptake; reported sexual risk for HIV and HIV screening uptake; and HIV screening uptake and an intersection of sexual risk for HIV (sex while intoxicated, regret ever having had sex while intoxicated and unsure if ever had sex while intoxicated) and alcohol misuse. There were some initial suggestions of a relationship between HIV screening uptake and the intersection of sexual risk for HIV and alcohol misuse, but demographic characteristics superseded this relationship.

These results raise questions as to why some relationships were found and not others. We observed a disconnection between sexual risk behaviors, alcohol misuse and HIV screening uptake. This finding suggests that participants in our study were unable to make crucial connections between their alcohol misuse and their sexual risk behaviors and translate this connection into a need for HIV testing. Based upon these results, we believe there is a need to reevaluate current alcohol misuse and HIV prevention and screening efforts that are being utilized in EDs. This disconnection among self-perceived, reported and actual risk and uptake of HIV screening has been observed in other studies [51,64,70,86-89]. For example, in a cross-sectional study conducted by MacKeller et al. in six US cities, 5,649 male participants who have sex with men were interviewed, were provided HIV sexual risk counseling and were offered HIV screening [90]. Of these participants, 77% of those that tested positive for HIV were unaware they were infected, 59% perceived themselves as low-risk for being infected with HIV and 44% perceived themselves as low-risk for ever becoming infected.

The need for effective interventions for the co-occurring problems of alcohol misuse and sexual risk for HIV in the ED is strongly suggested given the high-risk alcohol consumption and sexually risky behaviors reported by those in this study. A number of studies have demonstrated support for brief alcohol interventions in the ED [85,91]. However, we know of no published research examining sexual risk reduction interventions among ED patients. Furthermore, we know of no published research examining if a combination of brief alcohol interventions and HIV risk interventions is effective within this population in reducing sexual risk and increasing uptake of HIV screening. Support for this approach has been voiced by researchers in non-ED settings. Volkow et al. advocate that integrating substance abuse treatment into HIV prevention may improve public health outcomes (e.g. decreasing HIV incidence) and aid in reducing HIV transmission among injection and non-injection substance users [92]. In a randomized trial by Kalichman et al., 313 participants were randomly assigned to a three-hour HIV-alcohol risk-reduction skills intervention or a single one-hour HIV-alcohol education control group [93]. There was an increase of 100% usage of condoms or absence of sex in the lighter drinking group (77% vs. 43%; p < 0.01) but not in the heavier drinking group (55% vs. 59%, p < 0.05).

More research is needed to understand the connection between alcohol misuse, sexual risk for HIV and HIV screening uptake in the ED setting. Furthermore, patients who report high-risk behaviors, such as those identified in this study, for the acquisition of HIV may need help in recognizing these connections, reducing their risk behaviors, and accepting HIV testing. Further evaluations of the applicability and efficacy of integrated alcohol misuse and HIV sexual risk interventions within acute settings, such as EDs, is needed to determine effectiveness for this population. Intervention content regarding sexual risk behaviors in relation to alcohol misuse for ED patients should be evaluated and tested to reduce sexual risk and alcohol misuse and increase HIV screening uptake.

### Limitations

This study had a number of limitations. Self-report data regarding alcohol consumption and sexual risk for HIV may be inaccurate. Study participants may have underestimated or not recalled information regarding their alcohol consumption and HIV testing history. However, self-report of alcohol consumption and sexual behavior can be a reasonable method of obtaining these data [94,95]. Also, we did not collect data on whether or not the participant’s ED visit was related to their alcohol use. We do provide information regarding their level of at-risk drinking. Social desirability factors may have influenced some patients in their responses to reasons for accepting or declining screening, rather than any perception of their risk. Furthermore, it is unclear whether acceptance of screening based on an opt-out approach in the ED would be similar for participants who were excluded from the study. However, an opt-out approach may not be appropriate for patients who are unable to provide study consent. The HIV Sexual Risk Questionnaire has not been validated as a predictor of acquisition of HIV. As such, the true relationship between reported risk and HIV acquisition cannot be determined by this study. In addition, only 15.2% of women and 29.3% of men reported having unprotected sex with a casual partner (with or without an exchange or main partner), and most participants reported only sex with a main sexual partner. As such, the majority of participants could potentially be considered at lower risk for acquiring HIV, which might have appropriately influenced the uptake of testing. The small sample size may have produced limitations in identifying differences when they do exist. The study outcomes may not be appropriate for other EDs with different demographic characteristics, even though we attempted to obtain a representative sample by randomly selecting dates, shifts and participants. Of course, a failure to demonstrate relationships, should they exist, could have been related to the nature of the questions asked, their format, and the interpretation of the questions by the participants. Alternative questions, topics, and approaches could yield different results.

## Conclusions

Although there was a relationship between reported alcohol misuse and sexual risk for HIV, there appeared to be a disconnection between reported alcohol misuse, sexual risk for HIV and HIV screening uptake. Perhaps illustrating the connection between alcohol misuse and sexual risk within a brief intervention may create the opportunity for patients to recognize their level of risk, the connection between alcohol misuse and HIV risk behavior, and increase uptake of HIV screening in the ED and aid in reducing the prevalence of HIV within this high-risk population.

## Competing interests

The authors declare that they have no competing interests. This research was supported by grants from the National Institute of Drug Abuse (3R01 DA026066-02S1 of the American Recovery and Reinvestment Act and 3R01 DA026066-02S2 of the Research Supplements to Promote Diversity in Health-related Research Programs) and the Lifespan/Tufts/Brown Center for AIDS Research (P30 AI042853).

## Authors’ contributions

ADT performed the analyses for the study and prepared the manuscript. RCM, JRB, GTL and TDN were involved in the study design, execution, analysis and manuscript preparations. TL was involved in the biostatistical analyses and manuscript preparation. All authors read and approved the final manuscript.

## Pre-publication history

The pre-publication history for this paper can be accessed here:

http://www.biomedcentral.com/1471-227X/13/9/prepub

## Supplementary Material

Additional file 1 **Questionnaires Used in the Study. a.** Demographic Characteristics. **b.** Alcohol Use and Misuse Questionnaire. **c.** Alcohol Use Disorders Identification Test (AUDIT). **d.** Intersection of Alcohol Misuse and Sexual risk for HIV Behaviors. **e.** HIV Sexual Risk Questionnaire. Click here for file
