# Supplementary material for: Interrelationship of alcohol misuse, HIV sexual risk and HIV screening uptake among emergency department patients
Source: BMC Emerg Med. 2013 May 30;13:9. doi: 10.1186/1471-227X-13-9 (PMC3686630; doi:10.1186/1471-227X-13-9)
Supplement: Additional file 1 — Questionnaires Used in the Study. a. Demographic Characteristics. b. Alcohol Use and Misuse Questionnaire. c. Alcohol Use Disorders Identification Test (AUDIT). d. Intersection of Alcohol Misuse and Sexual risk for HIV Behaviors. e. HIV Sexual Risk Questionnaire. [file 1471-227X-13-9-S1.docx]

**Interrelationship of alcohol misuse, HIV sexual risk and HIV screening uptake among emergency department patients**

**Additional file 1**

1. **Questionnaires used in the study**
   1. Demographic Characteristics
   2. Alcohol Use Questionnaire
   3. Alcohol Use Disorders Identification Test (AUDIT)
   4. Conjunction of Alcohol Use/Misuse and HIV Sexual Risk
   5. HIV Sexual Risk Questionnaire

**Demographic Characteristics**

**1. What language would you prefer to speak?**

1. English
2. Spanish

**2. What is your age?**

**3. Do you consider yourself Hispanic/Latino?**

1. No
2. Yes

**4. Which one of these groups would you say best represents your race?**

1. White, Non-Hispanic 5. Asian
2. White, Hispanic 6. Native Hawaiian/Other Pacific Islander
3. Black, Non-Hispanic 7. American Indian/Alaskan Native
4. Black, Hispanic 8. Other

**5. What is the highest grade or year of school you completed?**

1. No School/ Kindergarten
2. Grades 1-8 (Elementary)
3. Grades 9-11 (Some High School)
4. Grade 12 or GED
5. College 1-3 years (Some College)
6. College 4 years (College grad)/ More Than College

**6. Do you have health insurance? What kind do you have? And is that through an employer or a spouse’s employer? Or is it from the state?**

1. Private
2. Governmental
3. Private and Governmental
4. None

**7. Are you…?**

1. Married
2. Divorced
3. Widowed
4. Separated
5. Never Married
6. Unmarried Couple

**8. How much do you weigh? (Pounds)**

**9. How tall are you? (Inches)**

**Alcohol Use Questionnaire**

1. **Think of a typical MONTH in the past 12 months when you drank alcohol. How many days** **in that typical month did you drink alcohol?**

1. **Think of a typical DAY when you drank alcohol in the past 12 months. How many drinks containing alcohol did you have on that typical day?**

1. **Think about the past 12 months when you drank alcohol. What type of drink containing alcohol did you typically have?**
2. Beer
3. Lite Beer
4. Malt Liquor
5. Mixed Drink (cocktail)
6. Shot (Straight or on ice)
7. Wine
8. Wine Cooler
9. **Think of a typical month when you drank alcohol in the past 12 months. What was the largest number of drinks containing alcohol that you drank on one occasion in that typical month?**
10. **Think of a typical month when you drank alcohol in the past 12 months. How many days did you have 5 or more drinks containing alcohol on one occasion on that typical month? (Males)**
11. **Think of a typical month when you drank alcohol in the past 12 months. How many days did you have 4 or more drinks containing alcohol on one occasion on that typical month? (Females)**

**Alcohol Use Disorders Identification Test (AUDIT)**

1. **How often do you have a drink containing alcohol?**
2. Never
3. Monthly or less
4. 2-4 times a month
5. 2-3 times a week
6. 4 or more times a week
7. **How many drinks containing alcohol do you have on a typical day when drinking?**
8. 1 or 2

2. 3 or 4

1. 5 or 6
2. 7 to 9
3. 10 or more
4. **How often do you have 6 or more drinks on one occasion?**
5. Never
6. Less than monthly
7. Monthly
8. Weekly
9. Daily or almost daily
10. **During the past 12 months, how often have you found that you were not able to stop drinking once you had started?**
11. Never
12. Less than monthly
13. Monthly
14. Weekly
15. Daily or almost daily
16. **During the past 12 months, how often have you failed to do what was normally expected of you because of drinking?**
17. Never
18. Less than monthly
19. Monthly
20. Weekly
21. Daily or almost daily
22. **During the past 12 months, how often have you needed a drink in the morning after a heavy drinking session?**
23. Never
24. Less than monthly
25. Monthly
26. Weekly
27. Daily or almost daily
28. **During the past 12 months, how often have you felt guilt after drinking?**
29. Never
30. Less than monthly
31. Monthly
32. Weekly
33. Daily or almost daily
34. **During the past 12 months, have you been unable to remember what happened the night before because you had been drinking?**
35. Never
36. Less than monthly
37. Monthly
38. Weekly
39. Daily or almost daily
40. **Have you or someone else been injured because of your drinking?**
41. No
42. Yes, but not in the last year
43. Yes during the last year
44. **Has a relative or friend, doctor or anyone else ever been concerned about your drinking or suggested that you cut down?**
45. No
46. Yes, but not in the last year
47. Yes during the last year

**Conjunction of Alcohol Use/Misuse and HIV Sexual Risk-Taking Behaviors**

1. **During the past 12 months, how often have you had sex only because you felt buzzed or drunk from alcohol?**

1. Every Time

2. Most of the Time

3. Some of the Time

4. Hardly Ever

5. Never

1. **During the past 12 months, how often did you have sex that you later regretted because you felt buzzed or drunk from alcohol?**

1. Every Time

2. Most of the Time

3. Some of the Time

4. Hardly Ever

5. Never

1. **During the past 12 months, how many times were you unsure if you had sex with someone because you felt buzzed or drunk from alcohol?**

1. Every Time

2. Most of the Time

3. Some of the Time

4. Hardly Ever

5. Never

**HIV Sexual Risk Questionnaire**

**FEMALES**

1. **In the past 12 months, have you had vaginal or anal sex with a man?**

1. No

2. Yes

1. **In the past 12 months, have you had any main partners? By main partners, we mean men you felt committed to, such as boyfriends, husbands,**

1. No

2. Yes

1. **Do you currently have a main male partner?**

1. No

2. Yes

1. **In the past 12 months, have you had any casual male partners? By casual partners, we mean you had sex with, but did not feel committed to.**

1. No

2. Yes

1. **In the past 12 months, have you had any exchange male partners? By exchange partners, we mean men you gave money, drugs, or other things to pay for sex, or men you had sex with so they would give you money, drugs, or other things.**

1. No

2. Yes

1. **In the past 12 months, have you had a sexually transmitted disease (STD) such as Chlamydia, gonorrhea, genital herpes or syphilis?**

1. No

2. Yes

1. **Please think about the times you had vaginal and/or anal sex with any of your main partners in the past 12 months. Has there ever been a time any of your main partners did not use a condom when having vaginal and/or anal sex with you?**

1. No, my main male partners always used a condom when having vaginal and/or anal sex with me in the past 12 months

2. Yes

1. **Please think about the main partners you had vaginal and/or anal sex with who did not use a condom. How many different main partners have you had vaginal and/or anal sex with in the past 12 months when they did not use a condom?**

1. 21 or More Different Men

2. 11-20 Different Men

3. 6-10 Different Men

4. 2-5 Different Men

5. 1 Man

1. **Please think about the main male partners you had vaginal and/or anal sex with in the past 12 months who did not use a condom. How many of these men do you KNOW had HIV or AIDS?**

1. 21 or More Different Men

2. 11-20 Different Men

3. 6-10 Different Men

4. 2-5 Different Men

5. 1 Man

6. None, I Know That **NONE** of These Men Had HIV/AIDS

1. **Please think about the main male partners you had vaginal and/or anal sex with in the past 12 months who did not use a condom. How many of these men are you UNSURE had HIV?**

1. 21 or More Different Men

2. 11-20 Different Men

3. 6-10 Different Men

4. 2-5 Different Men

5. 1 Man

6. DOES NOT APPLY, I KNOW that ALL of These Men Had HIV or AIDS

1. **Please think about the main male partners you had vaginal and/or anal sex with in the past 12 months who did not use a condom. How many of these men do you KNOW injected “street drugs”?**

1. 21 or More Different Men

2. 11-20 Different Men

3. 6-10 Different Men

4. 2-5 Different Men

5. 1 Man

6. None, I Know that **NONE** of These Men Injected “Street Drugs”

1. **Please think about the main male partners you had vaginal and/or anal sex with in the past 12 months who did not use a condom. How many of these men are you UNSURE if they injected “street drugs”?**

1. 21 or More Different Men

2. 11-20 Different Men

3. 6-10 Different Men

4. 2-5 Different Men

5. 1 Man

6. DOES NOT APPLY, I KNOW that ALL of These Men Injected “Street Drugs”

1. **Please think about the main male partners you had vaginal and/or anal sex with in the past 12 months who did not use a condom. How many of these men do you KNOW had a sexually transmitted disease (STD)?**

1. 21 or More Different Men

2. 11-20 Different Men

3. 6-10 Different Men

4. 2-5 Different Men

5. 1 Man

6. None, I know that **NONE** of These Men had a Sexually Transmitted Disease (STD)

1. **Please think about the main male partners you had vaginal and/or anal sex with in the past 12 months who did not use a condom. How many of these men are you UNSURE if he/they had a sexually transmitted disease (STD)?**

1. 21 or More Different Men

2. 11-20 Different Men

3. 6-10 Different Men

4. 2-5 Different Men

5. 1 Man

6. DOES NOT APPLY, I KNOW that ALL of These Men had a Sexually Transmitted Disease (STD)

1. **Please think about the main male partners you had vaginal and/or anal sex with in the past 12 months who did not use a condom. How many of these men do you KNOW ever had sex with other men?**

1. 21 or More Different Men

2. 11-20 Different Men

3. 6-10 Different Men

4. 2-5 Different Men

5. 1 Man

6. None, I know that **NONE** of These Men Ever had Sex with Men

1. **Please think about the main male partners you had vaginal and/or anal sex with in the past 12 months who did not use a condom. How many of these men are you UNSURE if they ever had sex with other men?**

1. 21 or More Different Men

2. 11-20 Different Men

3. 6-10 Different Men

4. 2-5 Different Men

5. 1 Man

6. DOES NOT APPLY, I KNOW that ALL of These Men had Sex with Other Men

1. **Please think about the main male partners you had vaginal and/or anal sex with in the past 12 months who did not use a condom. How many of these men did you have vaginal and/or anal sex with when you were feeling “buzzed” or drunk from alcohol?**

1. 21 or More Different Men

2. 11-20 Different Men

3. 6-10 Different Men

4. 2-5 Different Men

5. 1 Man

6. None

1. **Please think about the main male partners you had vaginal and/or anal sex with in the past 12 months who did not use a condom. How many of these men did you have vaginal and/or anal sex with when you were high, stoned or under the influence of drugs?**

1. 21 or More Different Men

2. 11-20 Different Men

3. 6-10 Different Men

4. 2-5 Different Men

5. 1 Man

6. None

1. **Please think about the times you had vaginal and/or anal sex with any of your casual male partners in the past twelve months. Has there ever been a time any of your casual partners did not use a condom when having vaginal and/or anal sex with you?**

1. No, My Casual Male Partners Always Used a Condom When Having Vaginal and/or Anal Sex with Me in the Past 12 Months

2. Yes

1. **Please think about the casual male partners you had vaginal and/or anal sex with who did not use a condom. How many different casual male partners have you had vaginal and/or anal sex with in the past 12 months when they did not use a condom?**

1. 21 or More Different Partners

2. 11-20 Different Partners

3. 6-10 Different Partners

4. 2-5 Different Partners

5. 1 Man

1. **Please think about the casual male partners you had vaginal and/or anal sex with in the past 12 months who did not use a condom. How many of these men do you KNOW had HIV or AIDS?**

1. 21 or More Different Partners

2. 11-20 Different Partners

3. 6-10 Different Partners

4. 2-5 Different Partners

5. 1 Man

6. None, I know that **NONE** of These Men had HIV or AIDS

1. **Please think about the casual male partners you had vaginal and/or anal sex with in the past 12 months who did not use a condom. How many of these men are you UNSURE had HIV or AIDS?**

1. 21 or More Different Partners

2. 11-20 Different Partners

3. 6-10 Different Partners

4. 2-5 Different Partners

5. 1 Man

6. DOES NOT APPLY, I KNOW that ALL of These Men had HIV or AIDS

1. **Please think about the casual male partners you had vaginal and/or anal sex with in the past 12 months who did not use a condom. How many of these men do you KNOW injected “street drugs”?**

1. 21 or More Different Men

2. 11-20 Different Men

3. 6-10 Different Men

4. 2-5 Different Men

5. 1 Man

6. None, I Know that **NONE** of These Men Injected “Street Drugs”

1. **Please think about the casual male partners you had vaginal and/or anal sex with in the past 12 months who did not use a condom. How many of these men are you UNSURE if they injected “street drugs”?**

1. 21 or More Different Men

2. 11-20 Different Men

3. 6-10 Different Men

4. 2-5 Different Men

5. 1 Man

6. DOES NOT APPLY, I KNOW that ALL of These Men Injected “Street Drugs”

1. **Please think about the casual male partners you had vaginal and/or anal sex with in the past 12 months who did not use a condom. How many of these men do you KNOW had a sexually transmitted disease (STD)?**

1. 21 or More Different Men

2. 11-20 Different Men

3. 6-10 Different Men

4. 2-5 Different Men

5. 1 Man

6. None, I know that **NONE** of These Men had a Sexually Transmitted Disease (STD)

1. **Please think about the casual male partners you had vaginal and/or anal sex with in the past 12 months who did not use a condom. How many of these men are you UNSURE if he/they had a sexually transmitted disease (STD)?**

1. 21 or More Different Men

2. 11-20 Different Men

3. 6-10 Different Men

4. 2-5 Different Men

5. 1 Man

6. DOES NOT APPLY, I KNOW that ALL of These Men had a Sexually Transmitted Disease (STD)

1. **Please think about the casual male partners you had vaginal and/or anal sex with in the past 12 months who did not use a condom. How many of these men do you KNOW ever had sex with other men?**

1. 21 or More Different Men

2. 11-20 Different Men

3. 6-10 Different Men

4. 2-5 Different Men

5. 1 Man

6. None, I know that **NONE** of These Men Ever had Sex with Men

1. **Please think about the casual male partners you had vaginal and/or anal sex with in the past 12 months who did not use a condom. How many of these men are you UNSURE if they ever had sex with other men?**

1. 21 or More Different Men

2. 11-20 Different Men

3. 6-10 Different Men

4. 2-5 Different Men

5. 1 Man

6. DOES NOT APPLY, I KNOW that ALL of These Men had Sex with Other Men

1. **Please think about the casual male partners you had vaginal and/or anal sex with in the past 12 months who did not use a condom. How many of these men did you have vaginal and/or anal sex with when you were feeling “buzzed” or drunk from school?**

1. 21 or More Different Men

2. 11-20 Different Men

3. 6-10 Different Men

4. 2-5 Different Men

5. 1 Man

6. None

1. **Please think about the casual male partners you had vaginal and/or anal sex with in the past 12 months who did not use a condom. How many of these men did you have vaginal and/or anal sex with when you were high, stoned or under the influence of drugs?**

1. 21 or More Different Men

2. 11-20 Different Men

3. 6-10 Different Men

4. 2-5 Different Men

5. 1 Man

6. None

1. **Please think about the times you had vaginal and/or anal sex with any of your exchange partners in the past 12 months. Has there ever been a time any of your exchange male partners did not use a condom when they were having vaginal and/or anal sex with you?**

1. No, My Exchange Male Partners Always Used a Condom When Having Vaginal and/or Anal Sex with Me in the Past 12 Months

2. Yes

1. **Please think about the exchange male partners you had vaginal and/or anal sex with who did not use a condom. How many different exchange male partners have you had vaginal and/or anal sex with in the past 12 months and they did not use a condom?**

1. 21 or More Different Partners

2. 11-20 Different Partners

3. 6-10 Different Partners

4. 2-5 Different Partners

5. 1 Man

1. **Please think about the exchange male partners you had vaginal and/or anal sex with in the past 12 months who did not use a condom. How many of these men do you KNOW had HIV or AIDS?**

1. 21 or More Different Partners

2. 11-20 Different Partners

3. 6-10 Different Partners

4. 2-5 Different Partners

5. 1 Man

6. None, I know that **NONE** of These Men had HIV or AIDS

1. **Please think about the exchange male partners you had vaginal and/or anal sex with in the past 12 months who did not use a condom. How many of these men are you UNSURE had HIV or AIDS?**

1. 21 or More Different Partners

2. 11-20 Different Partners

3. 6-10 Different Partners

4. 2-5 Different Partners

5. 1 Man

6. DOES NOT APPLY, I KNOW that ALL of These Men had HIV or AIDS

1. **Please think about the exchange male partners you had vaginal and/or anal sex with in the past 12 months who did not use a condom. How many of these men do you KNOW injected “street drugs”?**

1. 21 or More Different Men

2. 11-20 Different Men

3. 6-10 Different Men

4. 2-5 Different Men

5. 1 Man

6. None, I Know that **NONE** of These Men Injected “Street Drugs”

1. **Please think about the exchange male partners you had vaginal and/or anal sex with in the past 12 months who did not use a condom. How many of these men are you UNSURE if they injected “street drugs”?**

1. 21 or More Different Men

2. 11-20 Different Men

3. 6-10 Different Men

4. 2-5 Different Men

5. 1 Man

6. DOES NOT APPLY, I KNOW that ALL of These Men Injected “Street Drugs”

1. **Please think about the exchange male partners you had vaginal and/or anal sex with in the past 12 months who did not use a condom. How many of these men do you KNOW had a sexually transmitted disease (STD)?**

1. 21 or More Different Men

2. 11-20 Different Men

3. 6-10 Different Men

4. 2-5 Different Men

5. 1 Man

6. None, I know that **NONE** of These Men had a Sexually Transmitted Disease (STD)

1. **Please think about the exchange male partners you had vaginal and/or anal sex with in the past 12 months who did not use a condom. How many of these men are you UNSURE had a sexually transmitted disease (STD)?**

1. 21 or More Different Men

2. 11-20 Different Men

3. 6-10 Different Men

4. 2-5 Different Men

5. 1 Man

6. DOES NOT APPLY, I KNOW that ALL of These Men had a Sexually Transmitted Disease (STD)

1. **Please think about the exchange male partners you had vaginal and/or anal sex with in the past 12 months who did not use a condom. How many of these men do you KNOW ever had sex with other men?**

1. 21 or More Different Men

2. 11-20 Different Men

3. 6-10 Different Men

4. 2-5 Different Men

5. 1 Man

6. None, I know that **NONE** of These Men Ever had Sex with Men

1. **Please think about the exchange male partners you had vaginal and/or anal sex with in the past 12 months who did not use a condom. How many of these men are you UNSURE ever had sex with other men?**

1. 21 or More Different Men

2. 11-20 Different Men

3. 6-10 Different Men

4. 2-5 Different Men

5. 1 Man

6. DOES NOT APPLY, I KNOW that ALL of These Men had Sex with Other Men

**MALES**

1. **Please think about the exchange male partners you had vaginal and/or anal sex with in the past 12 months who did not use a condom. How many of these men did you have vaginal and/or anal sex with when you were feeling “buzzed” or drunk from alcohol?**

1. 21 or More Different Men

2. 11-20 Different Men

3. 6-10 Different Men

4. 2-5 Different Men

5. 1 Man

6. None

1. **Please think about the exchange male partners you had vaginal and/or anal sex with in the past 12 months who did not use a condom. How many of these men did you have vaginal and/or anal sex with when you were high, stoned or under the influence of drugs?**

1. 21 or More Different Men

2. 11-20 Different Men

3. 6-10 Different Men

4. 2-5 Different Men

5. 1 Man

6. None

1. **In the past 12 months, have you had vaginal and/or anal sex with a woman?**

1. No

2. Yes

1. **In the past 12 months, have you had any main female partners? By main partners, we mean women you felt committed to such as girlfriends, wives, significant others, or life partners.**

1. No

2. Yes

1. **Do you currently have a main female partner?**

1. No

2. Yes

1. **In the past 12 months, have you had any casual female partners? By casual partners, we mean women you had sex with, but did not feel committed to.**

1. No

2. Yes

1. **In the past 12 months, have you had any exchange female partners? By exchange partners, we mean women you gave money, drugs or other things to pay for sex, or women you had sex with so they would give you money, drugs or others things.**

1. No

2. Yes

1. **In the past 12 months, have you had a sexually transmitted disease (STD) such as Chlamydia, gonorrhea, genital herpes or syphilis?**

1. No

2. Yes

1. **Please think about the times you had vaginal and/or anal sex with any of your main female partners in the past 12 months. Has there ever been a time you did not use a condom when having vaginal and/or anal sex with any of your main female partners?**

1. No, I always used a condom when having vaginal and/or anal sex with my main female partner within the past 12 months

2. Yes

1. **Please think about those main female partners you had vaginal and/or anal sex with when you did not use a condom. How many different main female partners have you had vaginal and/or anal sex with in the past 12 months and you did not use a condom?**

1. 21 or More Different Women

2. 11-20 Different Women

3. 6-10 Different Women

4. 2-5 Different Women

5. 1 Woman

1. **Please think about the main female partners you had vaginal and/or anal sex with in the past 12 months when you did not use a condom. How many of these women do you KNOW had HIV or AIDS?**

1. 21 or More Different Women

2. 11-20 Different Women

3. 6-10 Different Women

4. 2-5 Different Women

5. 1 Woman

6. None, I know that **NONE** of These Women had HIV or AIDS

1. **Please think about the main female partners you had vaginal and/or anal sex with in the past 12 months when you did not use a condom. How many of these women are you UNSURE had HIV or AIDS?**

1. 21 or More Different Women

2. 11-20 Different Women

3. 6-10 Different Women

4. 2-5 Different Women

5. 1 Woman

6. DOES NOT APPLY, I KNOW that ALL of These Women had HIV or AIDS

1. **Please think about the main female partners you had vaginal and/or anal sex with in the past 12 months when you did not use a condom. How many of these women do you KNOW injected “street drugs”?**

1. 21 or More Different Women

2. 11-20 Different Women

3. 6-10 Different Women

4. 2-5 Different Women

5. 1 Woman

6. None, I know that **NONE** of These Women Injected Street Drugs

1. **Please think about the main female partners you had vaginal and/or anal sex with in the past 12 months when you did not use a condom. How many of these women are you UNSURE if they injected “street drugs”?**

21 or More Different Women

2. 11-20 Different Women

3. 6-10 Different Women

4. 2-5 Different Women

5. 1 Woman

6. DOES NOT APPLY, I KNOW that ALL of These Women Injected “Street Drugs”

1. **Please think about the main female partners you had vaginal and/or anal sex with in the past 12 months when you did not use a condom. How many of these women do you KNOW had a sexually transmitted disease (STD)?**

1. 21 or More Different Women

2. 11-20 Different Women

3. 6-10 Different Women

4. 2-5 Different Women

5. 1 Woman

6. None, I know that **NONE** of These Women had a Sexually Transmitted Disease (STD)

1. **Now think about the main partners you had vaginal and/or anal sex with in the past 12 months when you did not use a condom. How many of these women were you UNSURE if she/they had a sexually transmitted disease (STD)?**

1. 21 or More Different Women

2. 11-20 Different Women

3. 6-10 Different Women

4. 2-5 Different Women

5. 1 Woman

6. DOES NOT APPLY, I KNOW that ALL of These Women had a Sexually Transmitted Disease (STD)

1. **Please think about the main female partners you had vaginal and/or anal sex with in the past 12 months when you did not use a condom. How many of these women did you have vaginal and/or anal sex with when you were feeling “buzzed” or drunk from alcohol?**

1. 21 or More Different Women

2. 11-20 Different Women

3. 6-10 Different Women

4. 2-5 Different Women

5. 1 Woman

6. None

1. **Please think about the main female partners you had vaginal and/or anal sex with in the past 12 months when you did not use a condom. How many of these women did you vaginal and/or anal sex with when you were high, stoned or under the influence of drugs?**

1. 21 or More Different Women

2. 11-20 Different Women

3. 6-10 Different Women

4. 2-5 Different Women

5. 1 Woman

6. None

1. **Please think about the times you had vaginal and/or anal sex with any of your casual female partners in the past 12 months. Has there ever been a time you did not use a condom when having vaginal and/or anal sex with any of your casual female partners?**

1. No, I always used a condom when having vaginal and/or anal sex with my casual female partner within the past 12 months

2. Yes

1. **Please think about the casual female partners you had vaginal and/or anal sex with when you did not use a condom. How many different casual female partners have you had vaginal and/or anal sex with in the past 12 months and you did not use a condom?**

1. 21 or More Different Women

2. 11-20 Different Women

3. 6-10 Different Women

4. 2-5 Different Women

5. 1 Woman

1. **Please think about the casual female partners you had vaginal and/or anal sex with in the past 12 months when you did not use a condom. How many of these women do you KNOW had HIV or AIDS?**

1. 21 or More Different Women

2. 11-20 Different Women

3. 6-10 Different Women

4. 2-5 Different Women

5. 1 Woman

6. None, I know that **NONE** of These Women had HIV or AIDS

1. **Please think about the casual female partners you had vaginal and/or anal sex with in the past 12 months when you did not use a condom. How many of these women are you UNSURE had HIV or AIDS?**

1. 21 or More Different Women

2. 11-20 Different Women

3. 6-10 Different Women

4. 2-5 Different Women

5. 1 Woman

6. DOES NOT APPLY, I KNOW that ALL of These Women had HIV or AIDS

1. **Please think about the casual female partners you had vaginal and/or anal sex with in the past 12 months when you did not use a condom. How many of these women do you KNOW injected “street drugs”?**

1. 21 or More Different Women

2. 11-20 Different Women

3. 6-10 Different Women

4. 2-5 Different Women

5. 1 Woman

6. None, I know that **NONE** of These Women Injected Street Drugs

1. **Please think about the casual female partners you had vaginal and/or anal sex with in the past 12 months when you did not use a condom. How many of these women are you UNSURE if they injected “street drugs”?**

1. 21 or More Different Women

2. 11-20 Different Women

3. 6-10 Different Women

4. 2-5 Different Women

5. 1 Woman

6. DOES NOT APPLY, I KNOW that ALL of These Women Injected “Street Drugs”

1. **Please think about the casual female partners you had vaginal and/or anal sex with in the past 12 months when you did not use a condom. How many of these women do you KNOW had a sexually transmitted disease (STD)?**

1. 21 or More Different Women

2. 11-20 Different Women

3. 6-10 Different Women

4. 2-5 Different Women

5. 1 Woman

6. None, I know that **NONE** of These Women had a Sexually Transmitted Disease (STD)

1. **Please think about the casual female partners you had vaginal and/or anal sex with in the past 12 months when you did not use a condom. How many of these women are you UNSURE had a sexually transmitted disease (STD)?**

1. 21 or More Different Women

2. 11-20 Different Women

3. 6-10 Different Women

4. 2-5 Different Women

5. 1 Woman

6. DOES NOT APPLY, I KNOW that ALL of These Women had a Sexually Transmitted Disease (STD)

1. **Please think about the casual female partners you had vaginal and/or anal sex with in the past 12 months when you did not use a condom. How many of these women did you have vaginal and/or anal sex with when you were feeling “buzzed” or drunk from alcohol?**

1. 21 or More Different Women

2. 11-20 Different Women

3. 6-10 Different Women

4. 2-5 Different Women

5. 1 Woman

6. None

1. **Please think about the casual female partners you had vaginal and/or anal sex with in the past 12 months when you did not use a condom. How many of these women did you have vaginal and/or anal sex with when you were high, stoned or under the influence of drugs?**

1. 21 or More Different Women

2. 11-20 Different Women

3. 6-10 Different Women

4. 2-5 Different Women

5. 1 Woman

6. None

1. **Please think about the times you had vaginal and/or anal sex with any of your exchange female partners in the past 12 months. Has there ever been a time when you did not use a condom when having vaginal and/or anal sex with any of your exchange female partners?**

1. No, I always used a condom when having vaginal and/or anal sex with my exchange female partner within the past 12 months

2. Yes

1. **Please think about the exchange female partners you had vaginal and/or anal sex with when you did not use a condom. How many different exchange female partners have you had vaginal and/or anal sex with in the past 12 months and you did not use a condom?**

1. 21 or More Different Women

2. 11-20 Different Women

3. 6-10 Different Women

4. 2-5 Different Women

5. 1 Woman

1. **Please think about the exchange female partners you had vaginal and/or anal sex with in the past 12 months when you did not use a condom. How many of these women do you KNOW had HIV or AIDS?**

1. 21 or More Different Women

2. 11-20 Different Women

3. 6-10 Different Women

4. 2-5 Different Women

5. 1 Woman

6. None, I know that **NONE** of These Women had HIV or AIDS

1. **Please think about the exchange female partners you had vaginal and/or anal sex with in the past 12 months when you did not use a condom. How many of these women are you UNSURE had HIV or AIDS?**

1. 21 or More Different Women

2. 11-20 Different Women

3. 6-10 Different Women

4. 2-5 Different Women

5. 1 Woman

6. DOES NOT APPLY, I KNOW that ALL of These Women had HIV or AIDS

1. **Please think about the exchange female partners you had vaginal and/or anal sex with in the past 12 months when you did not use a condom. How many of these women do you KNOW injected “street drugs”?**

1. 21 or More Different Women

2. 11-20 Different Women

3. 6-10 Different Women

4. 2-5 Different Women

5. 1 Woman

6. None, I know that **NONE** of These Women Injected Street Drugs

1. **Please think about the exchange female partners you had vaginal and/or anal sex with in the past 12 months when you did not use a condom. How many of these women are you UNSURE if they injected “street drugs”?**

1. 21 or More Different Women

2. 11-20 Different Women

3. 6-10 Different Women

4. 2-5 Different Women

5. 1 Woman

6. DOES NOT APPLY, I KNOW that ALL of These Women Injected “Street Drugs”

1. **Please think about the exchange female partners you had vaginal and/or anal sex with in the past 12 months when you did not use a condom. How many of these women do you KNOW had a sexually transmitted disease (STD)?**

1. 21 or More Different Women

2. 11-20 Different Women

3. 6-10 Different Women

4. 2-5 Different Women

5. 1 Woman

6. None, I know that **NONE** of These Women had a Sexually Transmitted Disease (STD)

1. **Please think about the exchange female partners you had vaginal and/or anal sex with in the past 12 months when you did not use a condom. How many of these women are you UNSURE if she/they had a sexually transmitted disease (STD)?**

1. 21 or More Different Women

2. 11-20 Different Women

3. 6-10 Different Women

4. 2-5 Different Women

5. 1 Woman

6. DOES NOT APPLY, I KNOW that ALL of These Women had a Sexually Transmitted Disease (STD)

1. **Please think about the exchange female partners you had vaginal and/or anal sex with in the past 12 months when you did not use a condom. How many of these women did you have vaginal and/or anal sex with when you were feeling “buzzed” or drunk from alcohol?**

1. 21 or More Different Women

2. 11-20 Different Women

3. 6-10 Different Women

4. 2-5 Different Women

5. 1 Woman

6. None

1. **Please think about the exchange female partners you had vaginal and/or anal sex with in the past 12 months when you did not use a condom. How many of these women did you have vaginal and/or anal sex with when you were high, stoned or under the influence of drugs?**

1. 21 or More Different Women

2. 11-20 Different Women

3. 6-10 Different Women

4. 2-5 Different Women

5. 1 Woman

6. None

1. **In the past 12 months, have you had anal sex with a man?**

1. No

2. Yes

1. **In the past 12 months, have you had any main male partners? By main partners, we mean men you felt committed to, such as boyfriends, husbands, significant others, or life partners.**

1. No

2. Yes

1. **Do you currently have a main male partner?**

1. No

2. Yes

1. **In the past 12 months, have you had any casual male partners? By casual partners, we mean men you had sex with, but did not feel committed to.**

1. No

2. Yes

1. **In the past 12 months, have you had any exchange male partners? By exchange partners, we mean men you gave money, drugs, or other things to pay for sex, or men you had sex with so they would give you money, drugs, or other things.**

1. No

2. Yes

1. **In the past 12 months, have you had a sexually transmitted disease (STD) such as Chlamydia, gonorrhea, genital herpes or syphilis?**

1. No

2. Yes

1. **Please think about the times you had anal sex with any of your main male partners in the past 12 months. Has there ever been a time when you were the TOP when you did not use a condom when having anal sex with any of your main male partners?**

1. No, I always used a condom when I was the TOP with my main male partners

2. No, I was never the TOP in the past twelve months with my main male partners

3. Yes

1. **Please think about the main male partners you had anal sex with when you were the TOP and did not use a condom. How many different main male partners did you have anal sex with in the past 12 months when you were the TOP and did not use a condom?**

1. 21 or More Different Men

2. 11-20 Different Men

3. 6-10 Different Men

4. 2-5 Different Men

5. 1 Man

1. **Please think about the main male partners you had anal sex with in the past 12 months when you were the TOP and did not use a condom. How many of these men do you KNOW had HIV or AIDS?**

1. 21 or More Different Men

2. 11-20 Different Men

3. 6-10 Different Men

4. 2-5 Different Men

5. 1 Man

6. None, I know that **NONE** of these men had HIV or AIDS

1. **Please think about the main male partners you had anal sex with in the past 12 months when you were the TOP and did not use a condom. How many of these men are you UNSURE had HIV or AIDS?**

1. 21 or More Different Men

2. 11-20 Different Men

3. 6-10 Different Men

4. 2-5 Different Men

5. 1 Man

6. DOES NOT APPLY, I KNOW that ALL these men had HIV or AIDS

1. **Please think about the main male partners you had anal sex with in the past 12 months when you were the TOP and did not use a condom. How many of these men do you KNOW injected “street drugs”?**

1. 21 or More Different Men

2. 11-20 Different Men

3. 6-10 Different Men

4. 2-5 Different Men

5. 1 Man

6. None, I know that **NONE** of these men injected street drugs

1. **Please think about the main male partners you had anal sex with in the past 12 months when you were the TOP and did not use a condom. How many of these men are you UNSURE if they injected “street drugs”?**

1. 21 or More Different Men

2. 11-20 Different Men

3. 6-10 Different Men

4. 2-5 Different Men

5. 1 Man

6. DOES NOT APPLY, I KNOW that ALL these men had injected “street drugs”

1. **Please think about the main male partners you had anal sex with in the past 12 months when you were the TOP and did not use a condom. How many of these men do you KNOW had a sexually transmitted disease (STD)?**

1. 21 or More Different Men

2. 11-20 Different Men

3. 6-10 Different Men

4. 2-5 Different Men

5. 1 Man

6. None, I know that **NONE** of these men had a sexually transmitted disease (STD)

1. **Please think about the main male partners you had anal sex with in the past 12 months when you were the TOP and did not use a condom. How many of these men are you UNSURE had a sexually transmitted disease (STD)?**

1. 21 or More Different Men

2. 11-20 Different Men

3. 6-10 Different Men

4. 2-5 Different Men

5. 1 Man

6. DOES NOT APPLY, I KNOW that ALL of these men had a sexually transmitted disease (STD)

1. **Please think about the main male partners you had anal sex with in the past 12 months when you were the TOP and did not use a condom. How many of these men did you have anal sex with when you were feeling “buzzed” or drunk from alcohol?**

1. 21 or More Different Men

2. 11-20 Different Men

3. 6-10 Different Men

4. 2-5 Different Men

5. 1 Man

6. None

1. **Please think about the main male partners you had anal sex with in the past 12 months when you were the TOP and did not use a condom. How many of these men did you have anal sex with when you were high, stoned or under the influence of drugs?**

1. 21 or More Different Men

2. 11-20 Different Men

3. 6-10 Different Men

4. 2-5 Different Men

5. 1 Man

6. None

1. **Please think about the times you had anal sex with any of your casual male partners in the past 12 months. Has there ever been a time when you were the TOP when you did not use a condom when having anal sex with any of your casual male partners?**

1. No, I always used a condom when I was the TOP with my casual male partners

2. No, I was never the TOP in the past twelve months when I was with my casual male partners

3. Yes

1. **Please think about the casual male partners you had anal sex with when you were the TOP and did not use a condom. How many different causal partners did you have anal sex with in the past 12 months when you were the TOP and did not use a condom?**

1. 21 or More Different Men

2. 11-20 Different Men

3. 6-10 Different Men

4. 2-5 Different Men

5. 1 Man

1. **Please think about the casual male partners you had anal sex with in the past 12 months when you were the TOP and did not use a condom. How many of these men do you KNOW had HIV or AIDS?**

1. 21 or More Different Men

2. 11-20 Different Men

3. 6-10 Different Men

4. 2-5 Different Men

5. 1 Man

6. None, I know that **NONE** of these men had HIV or AIDS

1. **Please think about the exchange male partners you had anal sex with in the past 12 months when you were the TOP and did not use a condom. How many of these men are you UNSURE had HIV or AIDS?**

1. 21 or More Different Men

2. 11-20 Different Men

3. 6-10 Different Men

4. 2-5 Different Men

5. 1 Man

6. DOES NOT APPLY, I KNOW that ALL these men had HIV or AIDS

1. **Please think about the exchange male partners you had anal sex with in the past 12 months when you were the TOP and did not use a condom. How many of these men do you KNOW injected “street drugs”?**

1. 21 or More Different Men

2. 11-20 Different Men

3. 6-10 Different Men

4. 2-5 Different Men

5. 1 Man

6. None, I know that **NONE** of these men injected street drugs

1. **Please think about the exchange male partners you had anal sex with in the past 12 months when you were the TOP and did not use a condom. How many of these men are you UNSURE if they injected “street drugs”?**

1. 21 or More Different Men

2. 11-20 Different Men

3. 6-10 Different Men

4. 2-5 Different Men

5. 1 Man

6. DOES NOT APPLY, I KNOW that ALL these men injected “street drugs”

1. **Please think about the exchange male partners you had anal sex with in the past 12 months when you were the TOP and did not use a condom. How many of these men do you KNOW had a sexually transmitted disease (STD)?**

1. 21 or More Different Men

2. 11-20 Different Men

3. 6-10 Different Men

4. 2-5 Different Men

5. 1 Man

6. None, I know that **NONE** of these men had a sexually transmitted disease (STD)

1. **Please think about the exchange male partners you had anal sex with in the past 12 months when you were the TOP and did not use a condom. How many of these men are you UNSURE had a sexually transmitted disease (STD)?**

1. 21 or More Different Men

2. 11-20 Different Men

3. 6-10 Different Men

4. 2-5 Different Men

5. 1 Man

6. DOES NOT APPLY, I KNOW that ALL these men had a sexually transmitted disease (STD)

1. **Please think about the exchange male partners you had anal sex with in the past 12 months when you were the TOP and did not use a condom. How many of these men did you have anal sex with when you were feeling “buzzed” or drunk from alcohol?**

1. 21 or More Different Men

2. 11-20 Different Men

3. 6-10 Different Men

4. 2-5 Different Men

5. 1 Man

6. None

1. **Please think about the exchange male partners you had anal sex with in the past 12 months when you were the TOP and did not use a condom. How many of these men did you have anal sex with when you were high, stoned or under the influence of drugs?**

1. 21 or More Different Men

2. 11-20 Different Men

3. 6-10 Different Men

4. 2-5 Different Men

5. 1 Man

6. None

1. **Please think about the times you had anal sex with any of your main male partners in the past twelve months. Has there ever been a time when any of your main male partners did not use a condom when having anal sex with you when you were the BOTTOM?**

1. No, I was never the BOTTOM in the past twelve months with my main male partners

2. No, my main male partners always used a condom when I was the BOTTOM

3. Yes

1. **Please think about the main male partners you had anal sex with in the past 12 months when you were the BOTTOM and they did not use a condom. How many different main male partners did you have anal sex with in the past twelve months when you were the BOTTOM and they did not use a condom?**

1. 21 or More Different Men

2. 11-20 Different Men

3. 6-10 Different Men

4. 2-5 Different Men

5. 1 Man

1. **Please think about the main male partners you had anal sex with in the past 12 months when you were the BOTTOM and they did not use a condom. How many of these men do you KNOW had HIV or AIDS?**

1. 21 or More Different Men

2. 11-20 Different Men

3. 6-10 Different Men

4. 2-5 Different Men

5. 1 Man

6. None, I know that **NONE** of these men had HIV or AIDS

1. **Please think about the main male partners you had anal sex with in the past 12 months when you were the BOTTOM and they did not use a condom. How many of these men are you UNSURE had HIV or AIDS?**

1. 21 or More Different Men

2. 11-20 Different Men

3. 6-10 Different Men

4. 2-5 Different Men

5. 1 Man

6. DOES NOT APPLY, I KNOW that ALL these men had HIV or AIDS

1. **Please think about the main male partners you had anal sex with in the past 12 months when you were the BOTTOM and they did not use a condom. How many of these men do you KNOW injected “street drugs”?**

1. 21 or More Different Men

2. 11-20 Different Men

3. 6-10 Different Men

4. 2-5 Different Men

5. 1 Man

6. None, I know that **NONE** of these men injected “street drugs”

1. **Please think about the main male partners you had anal sex with in the past 12 months when you were the BOTTOM and they did not use a condom. How many of these men are you UNSURE if they injected “street drugs”?**

1. 21 or More Different Men

2. 11-20 Different Men

3. 6-10 Different Men

4. 2-5 Different Men

5. 1 Man

6. DOES NOT APPLY, I KNOW that ALL these men injected “street drugs”

1. **Please think about the main male partners you had anal sex with in the past 12 months when you were the BOTTOM and they did not use a condom. How many of these men do you KNOW had a sexually transmitted disease (STD)?**

1. 21 or More Different Men

2. 11-20 Different Men

3. 6-10 Different Men

4. 2-5 Different Men

5. 1 Man

6. None, I know that **NONE** of these men had a sexually transmitted disease (STD)

1. **Please think about the main male partners you had anal sex with in the past 12 months when you were the BOTTOM and they did not use a condom. How many of these men are UNSURE had a sexually transmitted disease (STD)?**

1. 21 or More Different Men

2. 11-20 Different Men

3. 6-10 Different Men

4. 2-5 Different Men

5. 1 Man

6. DOES NOT APPLY, I KNOW that ALL these men had a sexually transmitted disease (STD)

1. **Please think about the main male partners you had anal sex with in the past 12 months when you were the BOTTOM and they did not use a condom. How many of these men did you have anal sex with when you were feeling “buzzed” or drunk from alcohol?**

1. 21 or More Different Men

2. 11-20 Different Men

3. 6-10 Different Men

4. 2-5 Different Men

5. 1 Man

6. None

1. **Please think about the main male partners you had anal sex with in the past 12 months when you were the BOTTOM and they did not use a condom. How many of these men did you have anal sex with when you were high, stoned or under the influence of drugs?**

1. 21 or More Different Men

2. 11-20 Different Men

3. 6-10 Different Men

4. 2-5 Different Men

5. 1 Man

6. None

1. **Please think about the casual male partners you had anal sex with in the past 12 months when you were the BOTTOM and they did not use a condom. Has there ever been a time when any of your casual male partners did not use a condom when having anal sex with you when you were the BOTTOM?**

1. No, my casual male partners always used a condom when I was the BOTTOM

2. No, I was never the BOTTOM in the past twelve months with my casual partners

3. Yes

1. **Please think about the casual male partners you had anal sex with when you were the BOTTOM and they did not use a condom. How many different casual male partners did you have anal sex with in the past 12 months when you were the BOTTOM and they did not use a condom?**

1. 21 or More Different Men

2. 11-20 Different Men

3. 6-10 Different Men

4. 2-5 Different Men

5. 1 Man

1. **Please think about the casual male partners you had anal sex with in the past 12 months when you were the BOTTOM and they did not use a condom. How many of these men do you KNOW had HIV or AIDS?**

1. 21 or More Different Men

2. 11-20 Different Men

3. 6-10 Different Men

4. 2-5 Different Men

5. 1 Man

6. None, I know that **NONE** of these men had HIV or AIDS

1. **Please think about the casual male partners you had anal sex with in the past 12 months when you were the BOTTOM and they did not use a condom. How many of these men are you UNSURE had HIV or AIDS?**

1. 21 or More Different Men

2. 11-20 Different Men

3. 6-10 Different Men

4. 2-5 Different Men

5. 1 Man

6. DOES NOT APPLY, I KNOW that ALL these men had HIV or AIDS

1. **Please think about the casual male partners you had anal sex with in the past 12 months when you were the BOTTOM and they did not use a condom. How many of these men do you KNOW injected “street drugs”?**

1. 21 or More Different Men

2. 11-20 Different Men

3. 6-10 Different Men

4. 2-5 Different Men

5. 1 Man

6. None, I know that **NONE** of these men injected “street drugs”

1. **Please think about the casual male partners you had anal sex with in the past 12 months when you were the BOTTOM and they did not use a condom. How many of these men are you UNSURE if they injected “street drugs”?**

1. 21 or More Different Men

2. 11-20 Different Men

3. 6-10 Different Men

4. 2-5 Different Men

5. 1 Man

6. DOES NOT APPLY, I KNOW that ALL these men injected “street drugs”

1. **Please think about the casual male partners you had anal sex with in the past twelve months when you were the BOTTOM and they did not use a condom. How many of these men did you have anal sex with when you were feeling “buzzed” or drunk from alcohol?**

1. 21 or More Different Men

2. 11-20 Different Men

3. 6-10 Different Men

4. 2-5 Different Men

5. 1 Man

1. **Please think about the casual male partners you had anal sex with in the past twelve months when you were the BOTTOM and they did not use a condom. How many of these men did you have anal sex with when you were high, stoned or under the influence of drugs?**

1. 21 or More Different Men

2. 11-20 Different Men

3. 6-10 Different Men

4. 2-5 Different Men

5. 1 Man

6. None

1. **Please think about the exchange male partners you had anal sex with in the past 12 months when you were the BOTTOM and they did not use a condom. Has there ever been a time when any of your exchange male partners did not use a condom when having anal sex with you when you were the BOTTOM?**

1. No, my exchange partner always used a condom when I was the BOTTOM

2. No, I was never the BOTTOM in the past twelve months with my exchange partners

3. Yes

1. **Please think about the exchange male partners you had anal sex with when you were the BOTTOM and they did not use a condom. How many different exchange male partners did you have anal sex with in the past 12 months when you were the BOTTOM and they did not use a condom?**

1. 21 or More Different Men

2. 11-20 Different Men

3. 6-10 Different Men

4. 2-5 Different Men

5. 1 Man

1. **Please think about the exchange male partners you had anal sex with in the past 12 months when you were the BOTTOM and they did not use a condom. How many of these men do you KNOW had HIV or AIDS?**

1. 21 or More Different Men

2. 11-20 Different Men

3. 6-10 Different Men

4. 2-5 Different Men

5. 1 Man

6. None, I know that **NONE** of these men had HIV or AIDS

1. **Please think about the exchange male partners you had anal sex with in the past 12 months when you were the BOTTOM and they did not use a condom. How many of these men are you UNSURE had HIV or AIDS?**

1. 21 or More Different Men

2. 11-20 Different Men

3. 6-10 Different Men

4. 2-5 Different Men

5. 1 Man

6. DOES NOT APPLY, I KNOW that ALL these men had HIV or AIDS

1. **Please think about the exchange male partners you had anal sex with in the past 12 months when you were the BOTTOM and they did not use a condom. How many of these men do you KNOW injected “street drugs”?**

1. 21 or More Different Men

2. 11-20 Different Men

3. 6-10 Different Men

4. 2-5 Different Men

5. 1 Man

6. None, I know that **NONE** of these men injected “street drugs”

1. **Please think about the exchange male partners you had anal sex with in the past 12 months when you were the BOTTOM and they did not use a condom. How many of these men are you UNSURE if they injected “street drugs”?**

1. 21 or More Different Men

2. 11-20 Different Men

3. 6-10 Different Men

4. 2-5 Different Men

5. 1 Man

6. DOES NOT APPLY, I KNOW that ALL these men injected “street drugs”

1. **Please think about the exchange male partners you had anal sex with in the past twelve months when you were the BOTTOM and they did not use a condom. How many of these men did you have anal sex with when you were feeling “buzzed” or drunk from alcohol?**

1. 21 or More Different Men

2. 11-20 Different Men

3. 6-10 Different Men

4. 2-5 Different Men

5. 1 Man

1. **Please think about the exchange male partners you had anal sex with in the past twelve months when you were the BOTTOM and they did not use a condom. How many of these men did you have anal sex with when you were high, stoned or under the influence of drugs?**

1. 21 or More Different Men

2. 11-20 Different Men

3. 6-10 Different Men

4. 2-5 Different Men

5. 1 Man

6. None
